# Sequence motifs recognized by the casposon integrase of *Aciduliprofundum boonei*

**DOI:** 10.1093/nar/gkz447

**Published:** 2019-05-22

**Authors:** Pierre Béguin, Yankel Chekli, Guennadi Sezonov, Patrick Forterre, Mart Krupovic

**Affiliations:** 1 Unité Biologie Moléculaire du Gène chez les Extrêmophiles, Institut Pasteur, 25–28 rue du Dr. Roux 75724 Paris cedex 15, France; 2 UMRS 1138 – Centre de Recherche des Cordeliers, Sorbonne Université, 15, rue de l'École de Médecine, 75006 Paris, France; 3 Institute for Integrative Biology of the Cell (I2BC), CEA, CNRS, Univ. Paris- Sud, Université Paris-Saclay, Gif-sur-Yvette cedex, Paris, France

## Abstract

Casposons are a group of bacterial and archaeal DNA transposons encoding a specific integrase, termed casposase, which is homologous to the Cas1 enzyme responsible for the integration of new spacers into CRISPR loci. Here, we characterized the sequence motifs recognized by the casposase from a thermophilic archaeon *Aciduliprofundum boonei*. We identified a stretch of residues, located in the leader region upstream of the actual integration site, whose deletion or mutagenesis impaired the concerted integration reaction. However, deletions of two-thirds of the target site were fully functional. Various single-stranded 6-FAM-labelled oligonucleotides derived from casposon terminal inverted repeats were as efficiently incorporated as duplexes into the target site. This result suggests that, as in the case of spacer insertion by the CRISPR Cas1–Cas2 integrase, casposon integration involves splaying of the casposon termini, with single-stranded ends being the actual substrates. The sequence critical for incorporation was limited to the five terminal residues derived from the 3′ end of the casposon. Furthermore, we characterize the casposase from *Nitrosopumilus koreensis*, a marine member of the phylum Thaumarchaeota, and show that it shares similar properties with the *A. boonei* enzyme, despite belonging to a different family. These findings further reinforce the mechanistic similarities and evolutionary connection between the casposons and the adaptation module of the CRISPR–Cas systems.

## INTRODUCTION

The adaptive immune CRISPR–Cas system possessed by most archaea and many bacteria has been extensively characterized (1–5). During the adaptation stage, segments of incoming virus or plasmid DNA, termed protospacers, are integrated into the host genome at a locus, termed CRISPR, consisting of a cluster of palindromic repeats separated by spacers derived from previous infecting DNAs. During the expression and interference stages, upon reoccurring infection by the same plasmid or virus, the CRISPR cluster is transcribed into a pre-crRNA, which is subsequently fragmented to generate guide RNAs that target a complex of Cas (CRISPR-associated) proteins to promote the destruction of homologous infecting nucleic acids. Various systems belonging to different types and subtypes carry out the generation of guide RNAs and the subsequent destruction of invading DNAs or RNAs (6). By contrast, the two components, termed Cas1 and Cas2, that are responsible for the acquisition of new spacers, are highly conserved in the vast majority of CRISPR–Cas systems. Thus, this adaptation module appears to predate the modules for generating guide RNAs and the associated interference machineries, which probably evolved several times independently (7–9).

The mechanism by which the Cas1–Cas2 complex integrates new spacers into the CRISPR locus has been extensively characterized (10–13). The pre-spacer is loaded on a dumbell-shaped heterohexamer consisting of two Cas1 dimers separated by a Cas2 dimer, and lies across the protein complex, whose dimension acts as a yardstick for the length of the spacer. The ends of the pre-spacer are splayed, with the 3′ end of either strand interacting with one of the Cas1 subunits at either end of the complex (14,15). Transesterification reactions ligate the 3′ ends of the pre-spacer on either side of a CRISPR repeat, upon which the two strands of the repeat are separated and find themselves as single-stranded gaps flanking the newly integrated spacer. The gaps are subsequently repaired by the DNA polymerase and the ligase of the host, which leads to the duplication of the repeat (10,11,13). A key feature of the system is that acquisition of new spacers occurs preferentially at the level of the first repeat of the cluster. Indeed, the Cas1–Cas2 complex interacts either directly (16,17) or by means of an IHF-mediated bending of the target DNA (12,18) with sequence determinants of the leader lying upstream of the CRISPR cluster. The reaction of spacer integration into the CRISPR loci was noted to display mechanistic similarities with the integration of retroviruses and certain DNA transposons encoding transposases of the DDE superfamily (19–21). However, Cas1 is not homologous or structurally related to the retroviral integrases and DDE transposases and displays a novel fold (22).

**Figure 1.**
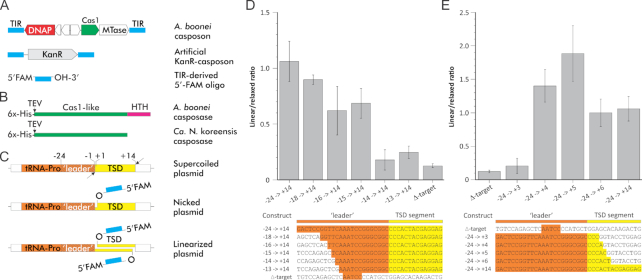
Schematic representation of the substrates used in the integration assays. (**A**) Genome maps of the *A. boonei* casposon and its derivatives: kanamycin resistance gene-carrying artificial casposon and 6-FAM-labeled oligonucleotide corresponding to the terminal inverted repeat (TIR) of the casposon. (**B**) Schematic representation of casposases from *A. boonei* and *Ca*. N. koreensis. The positions of the 6xHis-tag and the TEV protease cleavage site are indicated. The C-terminal helix-turn-helix domain of the *A. boonei* casposase is shown in magenta. (**C**) Schematic of the target site and the consequences of various integration intermediates on the topological state of the target site-carrying plasmids. (**D**) Effect of deletions shortening the leader segment of the *A. boonei* casposon. (**E**) Effect of deletions shortening the TSD segment. Plasmids harbouring the *A. boonei*-derived segments whose sequence is indicated under both graphs were incubated with the 6-FAM-labelled LE26 duplex (see Supplementary Table S1) and His-tagged Cas1 as described in Materials and Methods. The ratio between 6-FAM-labelled bands corresponding to the linearized and relaxed, circular forms of the plasmids was quantified for at least three independent reactions. Error bars indicate the standard deviation for each substrate. The sequence of the *A. boonei* segment corresponding to the TSD is shown in yellow and the upstream leader in orange. Numbering starts at the border between the leader sequence and the TSD, with +1 and –1 as the first TSD and the last leader nucleotides, respectively. Note that the depicted linear versus relaxed ratios should not be considered as actual numbers of single versus double integration events but rather compared with the control experiments.

A search across sequenced prokaryotic genomes for genes encoding homologs of CRISPR Cas1 led to the discovery of a new family of transposon-like mobile genetic elements termed casposons (23). As a defining common feature, casposons encode an integrase, termed casposase, which is homologous to the Cas1 subunit present in CRISPR–Cas systems. Yet, in casposons, the casposase gene is not associated with CRISPR loci nor with other *cas* genes, with a notable exception of *cas4*-like genes in some casposons (7). Instead, it is accompanied by a gene encoding a family B DNA polymerase and a variable set of other genes, such as DNA methyltransferases, nucleases, helicases as well as genes of unknown function (7,24). Similar to other transposons, casposons are bracketed between terminal inverted repeats (TIRs; Figure 1A), which are themselves flanked by direct repeats termed target site duplications (TSDs). TSDs are generated by a mechanism similar to that described in the CRISPR–Cas system for the generation of new repeats upon insertion of new spacers (7). Based on phylogenetic analysis of the casposases and gene content analysis, casposons are classified into four families (24). Notably, family 2 casposons contain a C-terminal helix-turn-helix domain, which is not found in casposases of the three other families or in CRISPR–Cas1 (Figure 1B) (7,23). Casposons from different families display distinct target site preferences, which broadly coincide with the casposase phylogeny. Most family 2 casposons are integrated in tRNA genes, family 3 and 4 casposons are found in the intergenic regions, whereas most family 1 casposons target 3′-distal regions of protein-coding genes (23,25).

The family 2 casposase encoded by a casposon of *Aciduliprofundum boonei*, a thermophilic archaeon, is currently the only experimentally characterized casposase. It has been purified and characterized *in vitro* after cloning and expression of its gene in recombinant *Escherichia coli* (21,26). The enzyme is able to perform the integration of an artificial casposon consisting of a kanamycin resistance gene flanked by the casposon-derived TIRs (Figure 1A). The reaction results in a 14–15-bp direct duplication of the target site (i.e. TSD) flanking the integrated casposon, indicating that integration proceeds via the same mechanism as the integration of spacers into CRISPR loci, which results in the duplication of the leader-proximal repeat (27). The enzyme can also catalyze the integration of oligonucleotides derived from the 3′ end of the casposon TIRs (Figure 1A). Single-end integration events on one strand of the target plasmid introduce a nick and lead to the relaxation of the supercoiled form of the plasmid, whereas concerted double-end integrations occurring at closely spaced, staggered sites on both strands linearize the plasmid (Figure 1C).

The *A. boonei* casposase seemed to display no detectable target site specificity when a generic plasmid such as pUC19 was used as a target (21). However, when a site reconstituting the original target sequence was available, integration occurred with high site preference, adjacent to and partially overlapping the tRNA-Pro gene of *A. boonei* (Figure 1C). Preliminary deletion mapping determined that motifs lying both within the TSD segment and in the upstream leader were necessary to form a functional target site (26).

In this study, we identified by deletion mapping and site-directed mutagenesis the sequence motifs that contribute to the recognition of the target by the *A. boonei* casposase. We also investigated which residues of the TIR 3′ end are required for efficient integration. Finally, we purified the family 1 casposase encoded by the casposon present in the genome of marine thaumarchaeon *Candidatus* Nitrosopumilus koreensis. The enzyme lacks the C-terminal helix-turn-helix domain present in the *A. boonei* casposase, yet its integrase activity displays properties very similar to those of the latter. The results further support the mechanistic similarities and evolutionary connections between casposons and the adaptation module of CRISPR–Cas adaptive immunity.

## MATERIALS AND METHODS

### Oligonucleotides

Oligonucleotides used for the creation of deletions or for site-directed mutagenesis were ordered from Sigma-Aldrich; 6-FAM-labelled oligonucleotides used in casposase assays were also from Sigma, except for the 6-FAM-labelled pentanucleotide LE5, which was ordered from Eurofins Genomics. Sequences of all oligonucleotides are listed in Supplementary Table S1.

### Target plasmids

The original pMA-Target plasmid carries a synthetic insert that comprises a single copy of the *A. boonei* TSD flanked on either side by the adjacent 125 bp of *A. boonei* DNA (26), which is cloned between a SacI and a KpnI site of the pMA vector (GeneArt). Deletions were constructed by PCR amplification using divergent primers flanking the region to be deleted and carrying either SacI or KpnI extensions, as described previously (Supplementary Figure S1 and Supplementary Table S1) (26). The amplified segments were gel-purified, treated with the corresponding enzymes plus DpnI to delete the original template, recircularized with T4 DNA ligase and transformed into TOP10 (Invitrogen) or NEB 10-β (New England Biolabs) chemically competent cells. A similar procedure was adopted to create point mutations: divergent PCRs were performed by combining a primer hybridizing to the vector next to the SacI or KpnI cloning site and another one carrying the desired mutation plus the corresponding restriction site extension and hybridizing to the target to be mutated. Likewise, amplified segments were digested with SacI or KpnI plus DpnI, recircularized and transformed. In order to avoid the need to use too long mutagenic oligonucleotides, mutants lying on the leader side of the target were created using pMA-Targ497-525, which carried the competent target with the shortest extension on the leader side (–15 to +14), and primers carrying SacI sites. Similarly, mutagenesis of sites located further downstream was performed using pMA-Targ488-517, which carried the competent target with the shortest downstream extension (–24 to +5), and primers carrying KpnI sites.

The plasmid pEX-A2-TSD-NitAR1 harbouring a synthetic insert reconstituting the integration target of the *Ca*. Nitrosopumilus koreensis casposon was ordered from GeneArt. The integration target comprises a single copy of the direct repeat (TSD) found on either side the casposon, flanked by 30 nucleotides derived from the genomic sequence lying upstream and downstream of the casposon.

### Production and purification of the casposases

Production, purification and removal of the His-tag by Tev protease from recombinant *A. boonei* casposase were performed as described previously (26) (Supplementary Figure S2A).

A codon-optimized version of the coding sequence of the *Ca*. Nitrosopumilus koreensis AR1 casposase (GenBank protein accession number: AFS80663; coded_by CP003842.1: 655943..656986) was ordered from GenArt™ (a subsidiary of Thermo Scientific). The gene, including the stop codon, was excised with *Pag*I and *Xho*I and recloned between the *Nco*I and *Xho*I sites of the pETM-11 vector (EMBL Protein Expression and Purification facility). Thus, the casposase was flanked at the NH_2_ terminus by the sequence MKHHHHHHPMSDYDIPTTENLYFQ, which provided a His_6_ affinity tag followed by a recognition site for Tev protease that enabled removal of the affinity tag (Figure 1B). The resulting plasmid, termed pETM11-NitAR1-Cas1, was introduced into the arabinose-inducible strain BL21AI (Invitrogen). The NitAR1 casposase was produced in 2YT medium containing 30 μg/ml kanamycin and 30 mg/ml chloramphenicol. Cultures of BL21AI(pETM11-NitAR1-Cas1) were grown up to an OD_600_ of 0.9–1, refrigerated to 14°C, induced with 1 mM IPTG and 0.2% l-arabinose and further incubated at 14°C overnight as described for the *A. boonei* casposase (26). Cell pellets from two 700-ml cultures in 2YT medium containing 30 μg/ml kanamycin were resuspended in 20 ml wash buffer (50 mM NaHPO_4_, pH 7.8, 0.4 M NaCl, 10 mM imidazole) containing 62,5 u/μl Benzonase® (Merck) and two freshly dissolved pellets of protease inhibitor cocktail (Roche cOmplete Ultra mini, EDTA-free). Cells were disrupted by two passages in a French press set at 100 MPa and the crude extract was centrifuged for 20 min at 40 000 g. The supernatant was loaded onto a 1.5 ml (bed volume) column of Talon Co^2+^ resin (Clontech) equilibrated with wash buffer. The column was rinsed extensively with wash buffer until the *A*_280_ of the effluent decreased <0.1 and the casposase was eluted with buffer containing 50 mM NaHPO_4_ pH 7.8, 0.4 M NaCl and 0.2 M imidazole. Fractions containing the protein were pooled and dialyzed against 50 mM Tris–HCl pH 8, 0.4 M NaCl.

To remove the His-tag, a 2-mg aliquot of purified casposase was treated for 25 h at 25°C with 0.4 mg His-tagged TEV protease in a final volume of 2 ml Tris–HCl 50 mM pH 8, 0.4 M NaCl, 1 mM dithiothreitol and 0.25 mM EDTA. Fifteen mM imidazole-HCl pH 8 was then added to the incubation mixture and the processed protein was separated from His-tagged polypeptides (TEV protease, unprocessed casposase, cleaved His-tag) by passage on a 1.5-ml Ni-NTA column (Qiagen) equilibrated with the same buffer. Unbound fractions were collected, concentrated by ultrafiltration and stored at –20°C after adding 50% (v/v) glycerol An SDS-polyacrylamide gel of the preparation is shown in Supplementary Figure S2B.

### Assays of the integration of labelled oligonucleotide substrates

To evaluate the competence of natural, partially deleted or mutated target segments, we performed integration assays with 6-FAM-labelled oligonucleotide duplexes corresponding to the 3′ termini of the *A. boonei* and *Ca*. N. koreensis casposons as described previously (26). Briefly, the target-bearing plasmids (1.5 ng/μl) were incubated with 200 nM 6-FAM-labelled duplex and 70 nM His-tagged casposase for 1 h at 37°C in 25 nM Tris–HCl pH 7.5 containing 150 mM KCl, 5 mM MnCl_2_ and 50 μg/ml bovine serum albumin. The reaction was stopped by adding 25 mM (final) Na-EDTA and treated for 1 h with 0.6 mg/ml proteinase K (Eurobio, 30 u/mg). Nucleic acids were ethanol precipitated, washed and taken up in 10 mM Tris–HCl pH 8, 1 mM EDTA before loading on an ethidium bromide-free 1% agarose gel. After completion of electrophoresis, the gels were scanned for 6-FAM fluorescence on a GE Typhoon FLA950 imager as described previously (26). We then determined the ratio of the relative intensities of the bands corresponding to integration into the relaxed or linear form of the plasmid using the ImageJ32 software (28). Results from at least three incubations were then processed using Kaleidagraph^®^ to yield the average ratio ± standard deviation of the two species.

The incorporation of various 6-FAM-labelled single-stranded oligonucleotides was performed similarly.

Restriction mapping of the concerted integration catalyzed by the casposase of *Ca*. N. koreensis was performed by digesting the pEX-A2-TSD-NitAR1 plasmid with ScaI (Thermo Fisher) or PagI (New England Biolabs). The mixture of fragments was column-purified using the Macherey Nagel Gel and PCR Clean-up kit, reacted with *Ca*. N. koreensis casposase and NitAR1-TR1 duplex (Supplementary Table S1) and analyzed as described above.

### Integration of an artificial casposon encoding kanamycin resistance

An artificial casposon encoding kanamycin resistance and flanked by the terminal inverted repeats present in the original casposon of *A. boonei* was incubated with the target plasmid pMA-Targ488-516, harbouring nucleotides –24 to +5 of the original target, as described previously (26). After transformation of the reaction mixture, plasmids were isolated from kanamycin-resistant clones and the insertion site of the casposon was determined by sequencing.

## RESULTS

### Mapping of motifs recognized by the *A. boonei* casposase in the integration target

To assess the functionality of various target sites, we performed an assay involving the integration of a labelled oligonucleotide duplex (LE26) derived from the 3′ end of the casposon TIR (Figure 1C). In a previous study (26), we noticed that when we used casposase bearing an N-terminal His-tag, efficient linearization resulting from concerted cleavage of both DNA strands of the target plasmid required the presence of the native target site of *A. boonei* (Figure 1C). Thus, we took advantage of this observation to identify motifs of the target whose deletion or mutation would reduce the intensity of labelling of the linear form relative to the relaxed form of the target plasmid. In a first step, we generated sets of deletions reaching either from the upstream (leader) or downstream side of the target region that we had previously circumscribed. Namely, the previously designed plasmid pMA-T103-140 (26) carried the shortest fragment still enabling efficient linearization, which comprised the leader sequence (positions –24 to –1) and the full TSD segment (positions +1 to +14) (Figure 1C). This plasmid was used as the template to further delete or systematically mutagenize individual positions and check which nucleotide residues were likely to be recognized by the casposase. The efficiency of integration was assessed as a ratio between the linearized (double-end integration) and relaxed (single-end integration) plasmid forms. Figure 1D shows that the linear/relaxed ratio dropped abruptly when deletions extended beyond nt -15 on the leader side. Remarkably, deletions that removed more than half of the TSD segment from the leader-distal side did not impair the tandem integration of the labelled oligonucleotide duplex LE26 (Figure 1E), indicating that the casposase shows little sequence specificity concerning the downstream processing site. For some unknown reason, the plasmid bearing the –24 to +5 target displayed an even higher linear versus relaxed ratio than plasmids carrying less extensive deletions within the TSD segment (Figure 1E).

**Figure 2.**
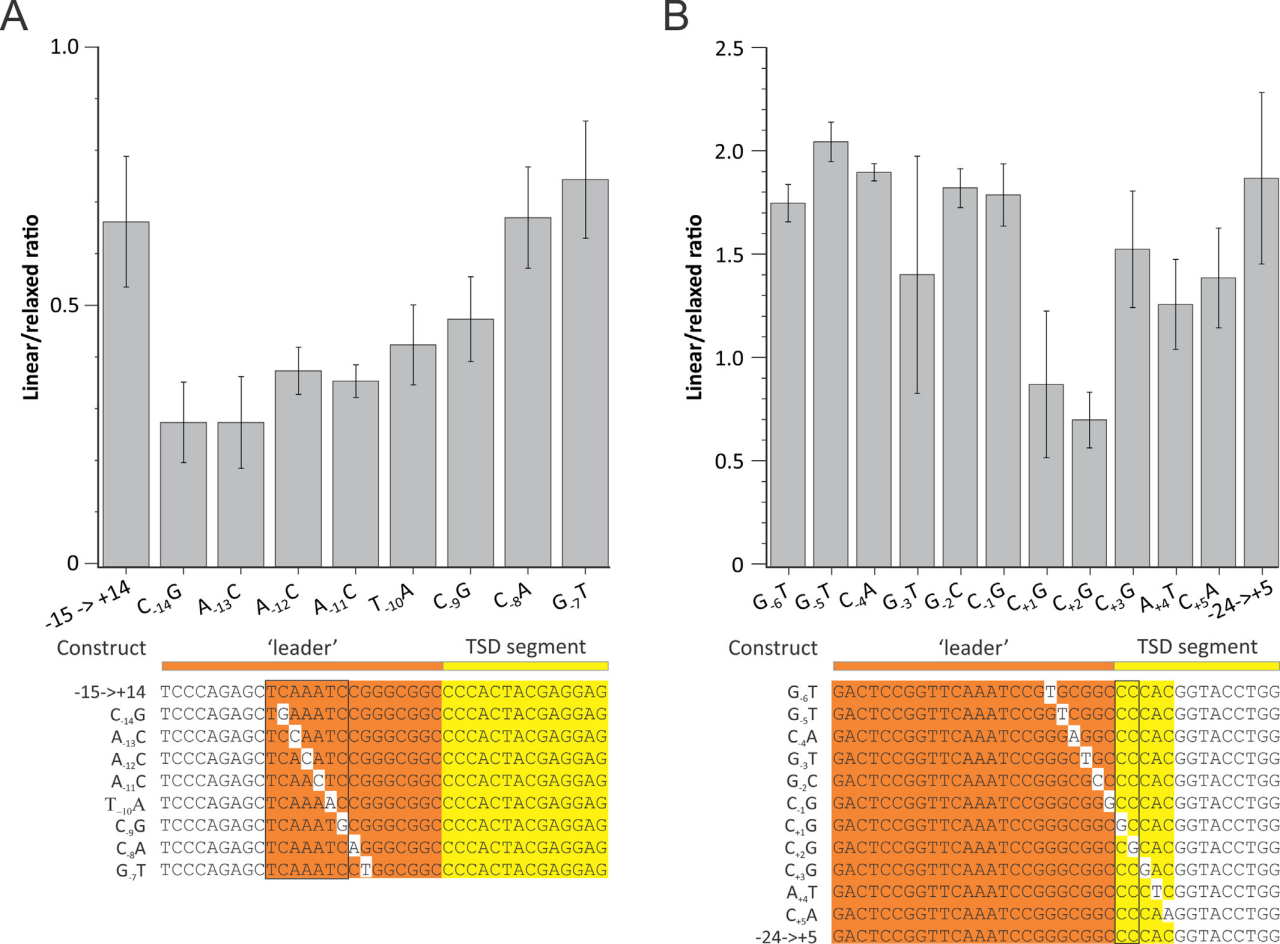
Effect of single nucleotide mutations affecting the target region of the *A. boonei* casposon. (**A**) Mutations generated from the ‘–15 to +14’ segment. B. Mutations generated from the ‘–24 to +5’ segment. Colours are the same as in Figure 1, with mutated residues shown on white background. Mutations affecting the linear:relaxed ratio most strongly are framed. Note that the depicted linear versus relaxed ratios should not be considered as actual numbers of single versus double integration events but rather compared with the control experiments.

To further identify the residues involved in target recognition, we selected the plasmids carrying the most extensive deletions on either side that would still be efficiently linearized and mutagenized every nucleotide position of the remaining segment. We then incubated the mutagenized plasmids with casposase and 6-FAM-labelled oligonucleotide duplex and monitored how the mutations affected the linear/relaxed ratio among the 6-FAM-labelled forms of the plasmid. Figure 2A shows that on the leader side, mutants of nucleotides –14 to –9 strongly impair linearization. Together with deletions carrying fragments –14 to + 14 and –13 to +14 (Figure 1D), these results suggest that the casposase recognizes the motif TCAAATC located in the leader region 8 bp upstream of the TSD segment. Mutants generated from the –24 to +5 target generally displayed a high linear versus relaxed ratio, similar to the parental plasmid, with the exception of mutants C_+1_G and C_+2_G, respectively, located 1 and 2 bp downstream of the leader-TSD border, respectively (Figure 2B). Thus, efficient casposon integration necessitates two sequence motifs, the 7-nt motif located within the leader sequence and a dinucleotide motif at the leader-TSD border.

Even though mutagenesis of critical nucleotides impaired the linearization of the plasmid, it did not affect the site preference of the casposase. Inasmuch as double-strand cleavage was detectable, restriction mapping of the reaction products indicated that it occurred at the target site (Supplementary Figure S3). This was further confirmed by performing the insertion of an artificial casposon carrying the kanamycin resistance gene into the plasmid harboring the –24 to +5 segment and sequencing the insertion site. When the reaction was performed with the His-tagged casposase, in 26 out of 28 clones tested the casposon was flanked by a duplication starting at nt +1 of the original target site and ending 14 bp downstream (Supplementary Figure S4). Integration using the de-tagged enzyme was less specific with only four out of 28 clones displaying insertion at the target site, consistent with the previous data showing that the integrations catalyzed by the de-tagged enzyme are more promiscuous (26).

### Insertion of single-stranded versus double-stranded TIR-derived oligos

**Figure 3.**
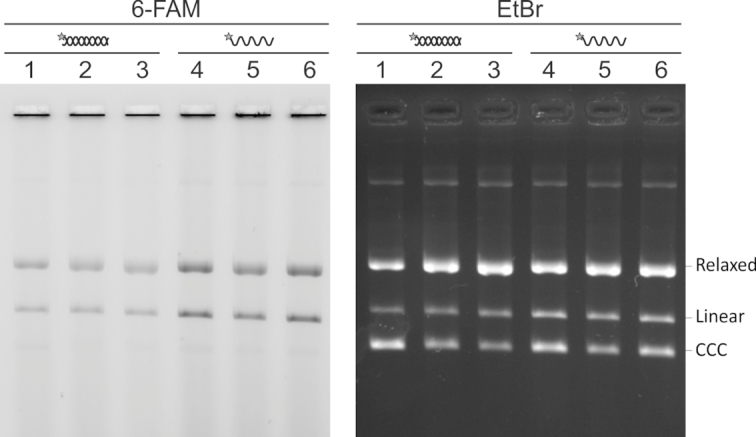
Comparison between the incorporation of double-stranded and single-stranded 6-FAM-labelled LE26 into pMA-Target. Three independent reactions were performed in parallel for either substrate. Lanes 1–3: 6-FAM-labelled LE26 duplex; lanes 4–6: single-stranded LE26. After quantifying the label incorporated into the linear and the relaxed form of the plasmid, the average total 6-FAM fluorescence of both forms observed with the single-stranded substrate was 7156 ± 309 arbitrary units for the duplex and 12878 ± 1501 for the single-stranded substrate. CCC, covalently closed circular DNA.

While trying to determine the shortest 6-FAM-labeled duplex that could be efficiently inserted into the target plasmid, we noticed that a 10-mer ‘duplex’ was incorporated as well or even better that the LE26 26-mer duplex, which was used in previous experiments (21,26). Since the incorporation reaction took place at 37°C, it was unlikely that the strands of a 10-mer duplex would remain hybridized, as its melting temperature was predicted to be <20°C. To confirm that the reaction would proceed with a single-stranded substrate, we compared, in triplicate, the incorporation of single-stranded and blunt-ended double-stranded LE26 (Tm = 58.2°C) into pMA-Target. Figure 3 shows that the single-stranded 6-FAM-labelled substrate was incorporated almost twice as efficiently as the duplex. This suggests that the process of casposon integration entails the splaying of the TIR within the active site of the casposase, such that the single-stranded 3′ end of the casposon is poised to undergo transesterification with the target DNA.

### Sequence requirements for the integration of TIR-derived oligonucleotides

**Figure 4.**
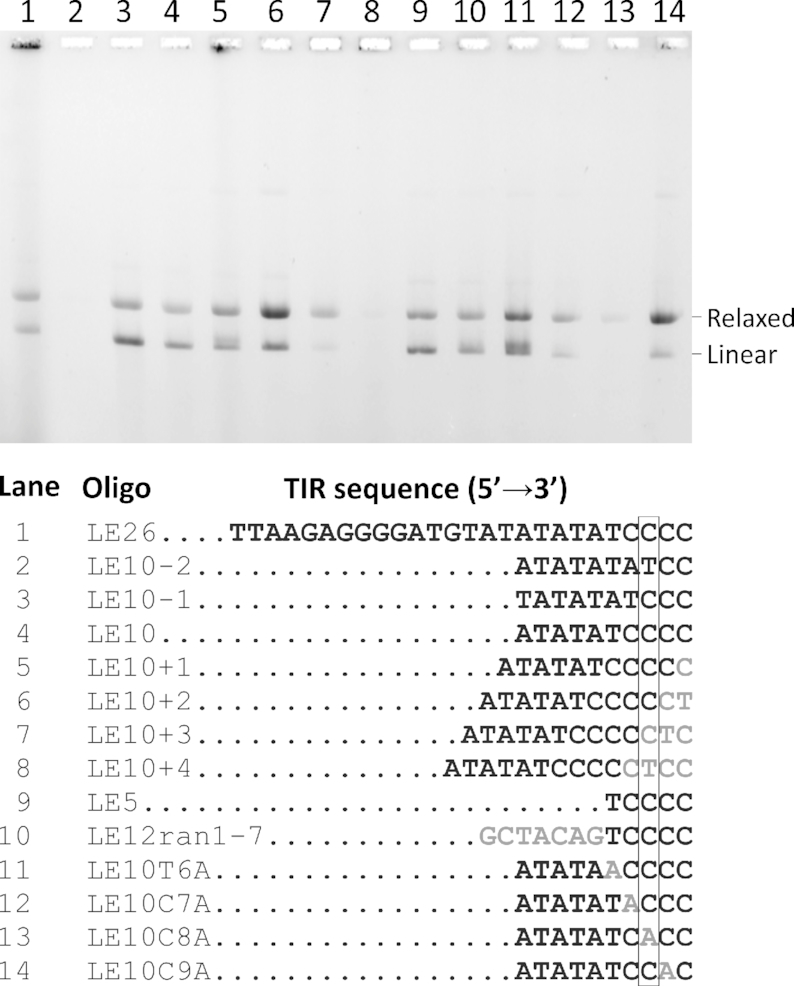
Insertion of various 6-FAM-labelled oligonucleotides derived from the left TIR of the *A. boonei* casposon into the plasmid pMA–Cas1–Target 488–525 carrying the –24 to +5 segment of the target. All oligonucleotides were labeled with 6-FAM in 5′. Except LE26, which was hybridized to the complementary strand, all oligonucleotides were single-stranded.

Hickman and Dyda showed that the insertion of oligonucleotides by *A. boonei* casposase was sequence-dependent, since an oligonucleotide of composition similar to that of the TIR, but with a scrambled sequence could not be integrated into the pUC19 plasmid (21). However, they also showed that the C present at the 3′ end of the TIR could be replaced by an A, indicating that substrate recognition involved subterminal residues rather than the 3′ end of the TIR (21). We set out to extend these observations by performing integration assays with different variants of the 3′ end of the TIR. Single-stranded substrates were used since they function at least as well as their double-stranded counterparts (see above). The oligonucleotides that were tested and the obtained incorporation patterns are shown in Figure 4. The shortest oligonucleotide tested, 6-FAM-LE5, a 5-mer with the sequence TCCCC, was efficiently incorporated (lane 9). Likewise, 6-FAM-LE12_ran_1-7, a 12-mer in which the first 7 nucleotides were scrambled relative to the TIR sequence, was also efficiently incorporated (lane 10), confirming that the nucleotides preceding the TCCCC motif don’t play a significant role in casposon end recognition. We then sought to determine which nucleotides of the TCCCC motif were important for efficient incorporation. Lanes 12 and 13 indicate that oligonucleotides in which the C_-4_ and particularly the C_–3_ nucleotides are exchanged for A are poorly incorporated, suggesting that these residues participate in enzyme-substrate recognition. T_–5_A and C_–2_A changes did not impair the incorporation (lanes 11 and 14). Another point was to find out how sensitive the enzyme was relative to the length of the 3′ end containing the TCCCC motif. One might expect that a too short substrate may not be able to reach the active site where transesterification is supposed to occur. Conversely, a too long extension may not work due to steric hindrance. The latter possibility had been previously shown by Hickman and Dyda for a 4-bp extension (21). Lanes 2–8 indicate that deletion of one or addition of up to two nucleotides to the canonical TCCCC motif are tolerated; addition of three nucleotides impairs the reaction partially; deletion of two or addition of four nucleotides abolish incorporation.

### Characteriation of a family 1 casposase from *Ca*. N. koreensis

**Figure 5.**
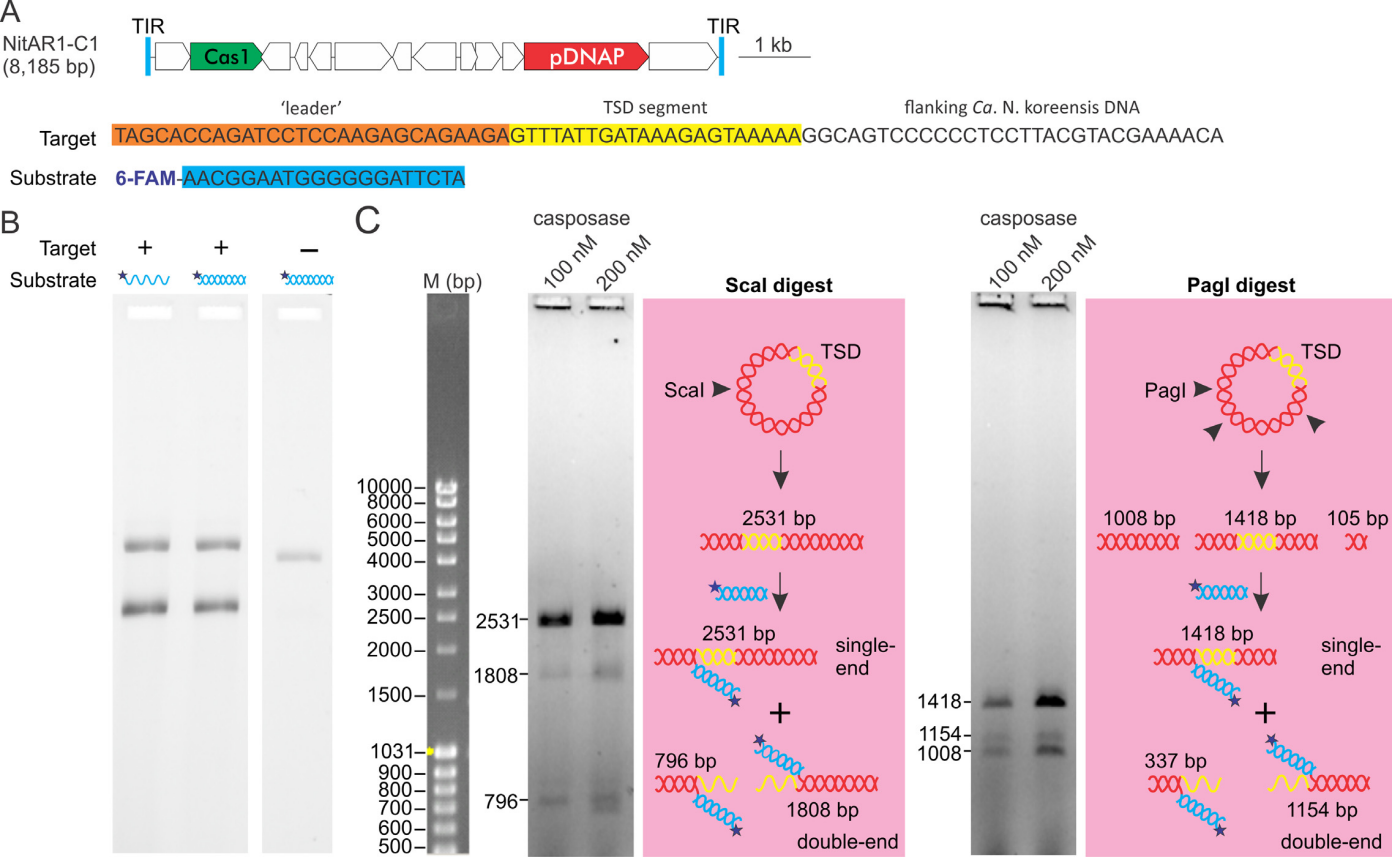
Characterization of the casposase of *Ca*. N. koreensis AR1. (**A**) Genome map of the NitAR1-C1 casposon. pDNAP, protein-primed family B DNA polymerase. The target sequence (leader sequence, orange; TSD segment, yellow) borne by the plasmid pEX-A2-TSD-NitAR1 and the TIR substrate NitAR1-TIR1 (cyan) derived from the TIR of the *Ca*. N. koreensis casposon used in the experiments are also shown. (**B**) Integration of double-stranded and single-stranded TIR-derived oligonucleotides. 100 nM of the de-tagged enzyme was incubated for 1 h at 37°C with 1.5 ng/μl plasmid bearing or not the reconstituted target segment and 200 nm of either single-stranded or double-stranded 6-FAM-labelled NitAR1-TIR1. (**C**) Site-specific integration of TIR-derived spacers into the target sequence. The plasmid carrying the target site was digested with ScaI, which linearizes the plasmid (2531 bp), or with PagI, which produces three linear fragments of 1418, 1008 and 105 bp. The resulting mixture of fragments was reacted with Cas1 and 6-FAM-labelled NitAR1-TIR1 duplex as described in Material and Methods. In the case of successful double-end integration of TIR-derived oligonucleotides into the target site, the target-bearing fragment is cleaved to produce lower molecular weight 6-FAM-labelled subfragments (see schematic). In the case of single-end integration the target-bearing fragments are 6-FAM-labeled but not cleaved. M, molecular size marker.

Until now, family 2 casposase from *A. boonei* was the only experimentally characterized casposase, raising a question of whether its properties can be generalized to casposases from other families. To address this point, we expressed and purified a family 1 casposase encoded by the NitAR1-C1 casposon (Figure 5A) integrated within the genome of a marine thaumarchaeon *Candidatus* Nitrosopumilus koreensis AR1 (23,25). Unlike the *A. boonei* enzyme, but similar to casposases from other families and CRISPR-associated Cas1, NitAR1-C1 casposase lacks the C-terminal HTH domain (Figure 1B). Also differently from *A. boonei* casposon, NitAR1-C1 is integrated into the 3′-distal region of a gene encoding elongation factor 2 (EF-2) We first tested if NitAR1 casposase can mediate concerted integration of TIR-derived 6-FAM-labeled double-stranded oligonucleotides into plasmids either containing or not the reconstituted target site (Figure 5A). Plasmid linearization resulting from a double-end integration was observed only in the presence of a target site, whereas incubation of the casposase with the plasmid lacking the cognate target site resulted in plasmid nicking and 6FAM-labeling, consistent with single-end integration event (Figure 5B). Next, to validate that plasmid linearization occurred at the target site, the target-bearing plasmids were digested with ScaI, which linearizes the plasmid (2531 bp), or PagI, which produces 3 linear fragments of 1418, 1008 and 105 bp. In the case of successful double-end integration of TIR-derived oligonucleotides into the target site, the target-bearing fragment is expected to be cleaved to produce lower molecular weight 6-FAM-labelled subfragments (796+1808 bp for ScaI, and 1154+337 bp for PagI; see schematic in Figure 5C). This experiment confirmed that plasmid linearization occurs specifically within the target bearing restriction fragments and generates subfragments of expected sizes. Finally, we tested if NitAR1 casposase, similar to *A. boonei* casposase, is capable of integrating single-stranded DNA oligonucleotides. Linearization of the target-bearing plasmid was comparable to that achieved with the double-stranded substrate (Figure 5B). Collectively, these results suggest that family 1 NitAR1 casposase shares similar properties with the *A. boonei* enzyme.

## DISCUSSION

We have previously demonstrated that the casposase encoded by the casposon of *A. boonei* shows a definite preference for a target site located at the 3′ end of the *tRNA-Pro* gene. The sequence required for a functional target was not limited to the TSD segment, i.e. the segment that got duplicated upon insertion of the casposon, but included also determinants located upstream of the TSD (26). This was highly reminiscent of the adaptation process of the CRISPR–Cas systems, where the integration of new spacers occurs next to the first palindromic repeat of the CRISPR locus and requires sequence determinants located within the leader segment lying upstream of the repeat cluster (16). Here, we provide further insights concerning substrate recognition by the *A. boonei* casposase. Mutations affecting target recognition are located within the segment TCAAATC, which lies 9–15 bp upstream of the casposon insertion site, and within the CC dinucleotide immediately following the upstream cleavage site of the TSD. These nucleotides are therefore suspected to make specific contacts with the enzyme. However, the interaction between casposase and the target DNA was too fleeting to be detected by gel shift assays upon incubating the DNA with up to 200 nm casposase; higher concentrations of enzyme led to precipitation, possibly induced by conformational changes of the casposase.

The physical separation between the sequence-specific recognition site and the catalytic site is not uncommon among nucleases and integrases. For example, site-specific insertion of Tn7 relies on the binding of the TnsD protein to a region that lies 22–55 bp from the site of insertion catalyzed by the TnsABC complex (29). Likewise, type IIs restriction enzymes such as FokI also feature a sequence recognition module that binds a sequence motif located 9 and 13 bp away from the cleavage sites on either DNA strands (30–32). It is also notable that certain transposons, such as members of the bacterial IS*630* and eukaryotic Tc/mariner families, recognize a dinucleotide (TA) in the target sequence (20,33,34), which might be equivalent to the CC motif recognized by the casposase. However, in the case of these transposons, the transesterification reactions occur on both sides of the dinucleotide, whereas in the case of the casposase, the second nucleophilic attack takes place 12 bp away from the dinucleotide motif.

Deletion of residues lying in the vicinity of the 3′ end of the TSD do not impair concerted integration: in the plasmid harbouring the –24 to +5 target, insertion at the 3′ site occurs in most cases 14 bp downstream of the 5′ site, indicating that the separation of the two sites is determined by a ‘yardstick’ provided by the distance between two active sites of the enzyme required for integrating both ends of the casposon. This is consistent with the data of Hickman and Dyda, who reported that casposon insertions at random sites of pUC19, which contains no target site, result in a 14–15 bp duplication at the insertion site (21). A similar feature has been described for the CRISPR type I-B adaptation system of *Haloarcula hispanica* (35), a euryarchaeon like *A. boonei*. In this organism, sequence motifs flanking the border between the first repeat and the leader are required for efficient integration and determine the position of the leader-proximal insertion site, whereas transesterification at the spacer-proximal site is sequence-independent and occurs at a fixed distance from an anchor motif located in the middle of the repeat.

Regarding the TIRs of the casposons, the fact that single-stranded oligonucleotides are incorporated into the target plasmids at least as efficiently as duplexes by both family 1 and family 2 casposases suggests that casposon integration entails the splaying of the termini of the incoming fragment and that this feature is general for this superfamily of enzymes. The structural analysis of the Cas1–Cas2 integrase co-crystallized with a protospacer indicates that the ends of the protospacer are indeed splayed (12,14,15). However, contrary to casposase, the Cas1–Cas2 integrase does not perform the integration of single-stranded substrates (36). This may be related to the fact that in the CRISPR–Cas system, the entire protospacer including the double-stranded middle segment, contacts the integration complex, which acts like a yardstick (14). By contrast, due to the length of the incoming casposon, interaction with the casposase is by necessity limited to the TIR. It may therefore not rely on extensive contact with the double-stranded region of the casposon.

The requirements regarding the sequence of the TIR were rather limited. The pentamer 6-FAM-TCCCC was efficiently incorporated by the *A. boonei* casposase, and adding two or removing one nucleotide from the 3′ end did not impair the reaction significantly. This tolerance may be explained if the enzyme allows some slippage of the oligo-C tract for the correct positioning of the reactive 3′ end. However, the C located at the -3 position appears essential: the insertion of the LE10C8A substrate was strongly impaired; this is also consistent with the poor insertion of the LE10-2 and LE10+4 oligonucleotides, which both feature a T instead of a C at the -3 position (Figure 4). The finding that only a short stretch of nucleotides in the 3′ end of the TIR is required for the efficient integration reaction compares with the near-absence of sequence signatures required for selective protospacer recruitment from the viral/plasmid DNA and integration into *E. coli* CRISPR loci. Indeed, protospacers are selected based on the presence of protospacer adjacent motifs (PAM), which typically consist of 2–5 nucleotides (14,27,37,38). Furthermore, it has been shown that CRISPR–Cas1 and PAM coevolve, indicating direct interaction between the two components (37). Indeed, structural studies have shown that the catalytic Cas1 subunit recognizes the PAM-complementary sequence in the 3′ overhang of the protospacer in a base-specific manner (14). Once complexed with the Cas1–Cas2 integrase, the PAM motif is trimmed away, an activity attributed to Cas4 nuclease in several type I systems (39–42), or to a DnaQ exonuclease domain fused to Cas2 (43). Given that the five 3′-terminal nucleotides of the TIR is the only casposon-carried determinant specifically required for casposon integration, the PAM recognition by the CRISPR–Cas1 and the TIR recognition by the casposase might be functionally equivalent, if not evolutionarily related. The main difference between the two systems is that the TIRs do not appear to be processed, although the information on casposon termini generated *in vivo* is still lacking. Notably, however, some casposons are known to encode Cas4-like nucleases (7), which may be involved in the processing of casposon termini. The sequence features required for efficient casposon integration reported here for the *A. boonei* casposon may be more broadly conserved among casposons. Previous comparative genomics analysis of casposons integrated in the genomes of *Methanosarcina mazei* strains showed that only the first four leader-proximal nucleotides of the 14-bp-long TSD were absolutely conserved, whereas other positions were variable (24).

Preliminary characterization of a family 1 casposase encoded by a casposon integrated in the genome of a mesophilic marine thaumarchaeon suggests that features of the more deeply studied *A. boonei* casposase can be generalized to casposases from other families. In particular, this concerns the target-specificity of the integration reaction and the ability to integrate single-stranded oligonucleotides derived from the casposon TIRs. Notably, NitAR1 casposase lacks the C-terminal HTH domain present in the *A. boonei* casposase, which makes it more similar to the CRISPR-associated Cas1 enzymes. Comparison of the family 1 and 2 casposases suggests that the C-terminal HTH domain of family 2 enzymes is not essential for catalysis but the actual role of this domain remains to be investigated.

Collectively, our results reinforce the similarities and evolutionary connection between the casposons and the adaptation module of the prokaryotic CRISPR–Cas systems. Recently, this connection was further supported by the discovery of a CRISPR–Cas system whose adaptation module consists of a Cas1 tetramer with no Cas2 subunit (44). At the same time, casposons and CRISPR–Cas systems fulfil different requirements, the former being to make copies of itself and insert them into the host genome and the latter to generate a library of DNA fragments derived from invading DNAs. At the moment, it is not known how casposons are duplicated and liberated from the host chromosome. Different transposition pathways have been described for transposons (for reviews see 45,46). In this respect, since only five terminal nucleotides of the TIR are required for casposon integration, the conservation of a 37-bp palindromic TIR suggests that it carries motifs required for other processes, such as excision or formation of replicative intermediates, as proposed previously (23). Such issues would deserve further investigations.

## Supplementary Material

gkz447_Supplemental_Files

## Notes

Present address: Yankel Chekli, Genetics of Biofilm Unit, Institut Pasteur, 25–28 rue du Dr. Roux, 75724 Paris cedex 15, France.
