# Supplementary material for: Sequence motifs recognized by the casposon integrase of Aciduliprofundum boonei
Source: Nucleic Acids Res. 2019 May 22;47(12):6386–95. doi: 10.1093/nar/gkz447 (PMC6614799; doi:10.1093/nar/gkz447)
Supplement: gkz447_Supplemental_Files [file gkz447_supplemental_files.pdf]

Supplementary Table 1. Oligonucleotides used in this study.

Set of primers for site-directed mutagenesis

<u>Mutagenesis using the *Sac*I site</u>

Name	Sequence	Deletion/ mutation
SacI-Targ388r	ATTGAGCTCTGGACAAGACACGTG	Rev. primer*
SacI-Targ494	AAAGAGCTCAGGTTCAAATCCGGGCGG	-18->+14
SacI-Targ496	TTGTCCAGAGCTCTTCAAATCCGGGCGG	-16->+14
SacI-Targ497	AAAGAGCTCAAATCCGGGCGGCC	-15->+14
SacI-Targ498	AAAGAGCTCGCAAATCCGGGCGGCC	-14->+14
SacI-Targ499	AAAGAGCTCGAAATCCGGGCGGCCC	-13->+14
SacI-TargT497G	TTGTCCAGAGCTCGAAAATCCGGGCGGCCCCA	T$_{-15}$G
SacI-TargC498G	TTGTCCAGAGCTCTGAAATCCGGGCGGCCCCA	C$_{-14}$G
SacI-TargA499C	TTGTCCAGAGCTCCAATCCGGGCGGCC	A$_{-13}$C
SacI-TargA500C	TTGTCCAGAGCTCACATCCGGGCGGCC	A$_{-12}$C
SacI-TargA501C	TTGTCCAGAGCTCAACTCCGGGCGGCC	A$_{-11}$C
SacI-TargT502A	TTGTCCAGAGCTCAAAACCGGGCGGCC	T$_{-10}$A
SacI-TargC503G	TTGTCCAGAGCTCAAATGCGGGCGGCC	C$_{-9}$G
SacI-TargC504A	TTGTCCAGAGCTCAAATCAGGGCGGCCC	C$_{-8}$A
SacI-TargG505T	CCAGAGCTCAAATCCTGGCGGCCCCCACT	G$_{-7}$T

<u>Mutagenesis using the *Kpn*I site</u>

Name	Sequence	Deletion/ mutation
KpnI-Targ648	AAAGGTACCTGGAGCACAAGACTGG	Rev. primer*
KpnI-TargG506T	AAAGGTACCGTGGGGCCGCACGGATTTGAAC	G$_{-6}$T
KpnI-TargG507T	AAAGGTACCGTGGGGCCGACCGGATTTGAAC	G$_{-5}$T
KpnI-TargC508A	AAAGGTACCGTGGGGCCTCCCGGATTTGAAC	C$_{-4}$A
KpnI-TargG509T	AAAGGTACCGTGGGGCAGCCCGGATTTGAAC	G$_{-3}$T
KpnI-TargG510C	CCAGGTACCGTGGGGGCGCCCGGATTTGA	G$_{-2}$C
KpnI-TargC511G	CCAGGTACCGTGGGCCCGCCCGGATTTGA	C$_{-1}$G
KpnI-TargC512G	TCCAGGTACCGTGGCGCCGCCCGGATTTG	C$_{+1}$G
KpnI-TargC513G	CTCCAGGTACCGTGCGGCCGCCCGGATTT	C$_{+2}$G
KpnI-TargC514G	GCTCCAGGTACCGTCGGGCCGCCCGGATT	C$_{-3}$G
KpnI-TargA515T	GCTCCAGGTACCGAGGGGCCGCCCGGATT	A$_{+4}$T
KpnI-Targ514r	AAAGGTACCGGGGCCGCCCGG	-24->+3
KpnI-Targ515r	AAAGGTACCTGGGGCCGCCCG	-24->+4
KpnI-Targ516r	AAAGGTACCGTGGGGCCGCCCG	-24->+5
KpnI-Targ517r	AAAGGTACCAGTGGGGCCGCCCG	-24->+6

* The reverse primer is the divergent primer used for amplification with the mutagenic primers. Mutagenic nucleotides are highlighted in yellow. See Supplementary Figure 1.

Set of primers derived from the TIR of the *A. boonei* casposon

Name	Sequence
LE26	6-FAM-TTAAGAGGGGATGTATATATATCCCC
LE26r	GGGGATATATATACATCCCCTCTTAA
LE10-2	6-FAM-ATATATATCC
LE10-1	6-FAM-TATATATCCC
LE10	6-FAM-ATATATCCCC
LE10+1	6-FAM-ATATATCCCCC
LE10+2	6-FAM-ATATATCCCCCT
LE10+3	6-FAM-ATATATCCCCCTC
LE10+4	6-FAM-ATATATCCCCCTCC
LE5	6-FAM-TCCCC
LE12ran1-7	6-FAM-GCTACAGTCCCC
LE10T6A	6-FAM-ATATAACCCC
LE10C7A	6-FAM-ATATATACCC
LE10C8A	6-FAM-ATATATCACC
LE10C9A	6-FAM-ATATATCCAC

Set of primers derived from the TIR of the *Ca.* N. koreensis NitAR1 casposon

NitAR1-TIR1	6-FAM-AACGGAATGGGGGGATTCTA
NitAR1-TIR1r	TAGAATCCCCCCATTCCGTT

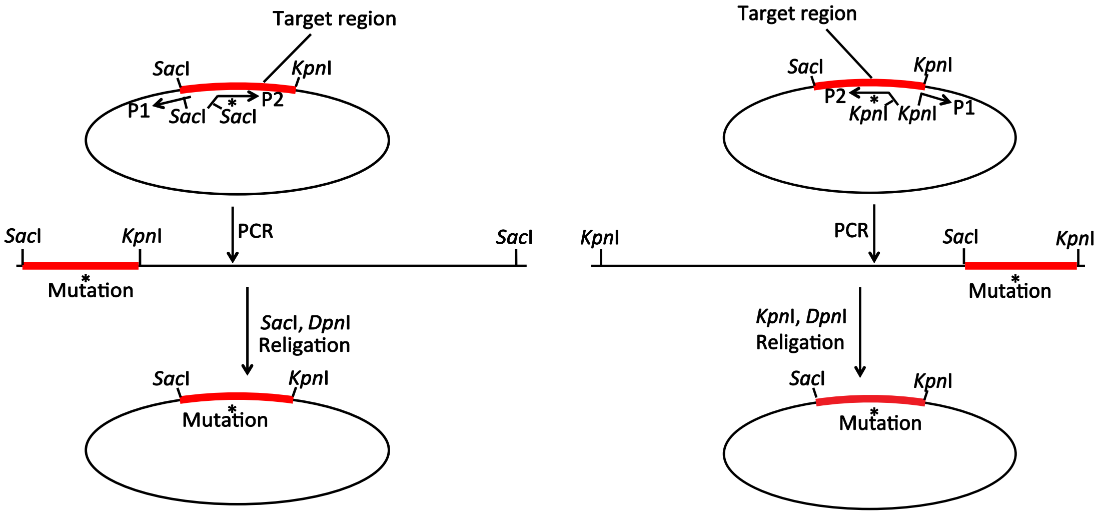

Supplementary Figure 1: Mutagenesis of the target sequence (in red) recognized by the Cas1 integrase. For each mutation, a PCR reaction was performed using an oligonucleotide hybridizing to the vector-insert border and including a 5' SacI or 5' KpnI site and another oligonucleotide comprising the desired mutation (*) plus the same restriction site as the first one. The resulting linear fragment was digested with SacI or KpnI to generate cohesive ends, and with DpnI to destroy the wild type template. Re-ligation yielded a circular plasmid ready for transformation and carrying the mutated target site.

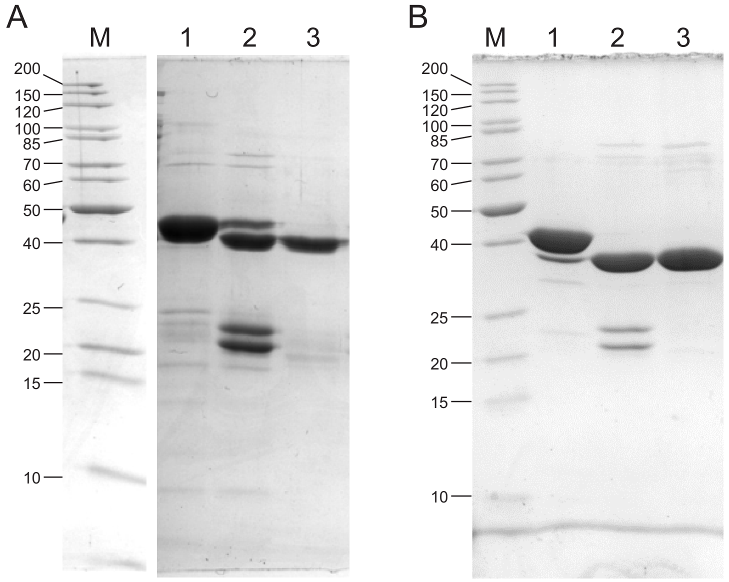

Supplementary Figure 2: Purification of *A. boonei* (A) and *Ca*. N. koreensis (B) casposases. Lane 1, His-tagged purified fraction after Ni-NTA chromatography of the crude extract; lane 2, mixture of casposase and His-tagged TEV protease after overnight treatment at 25 °C; lane 3, de-tagged casposase recovered in the fraction not retained on Ni-NTA resin after TEV protease treatment; M molecular size (kDa) marker (Fermentas PageRuler unstained).

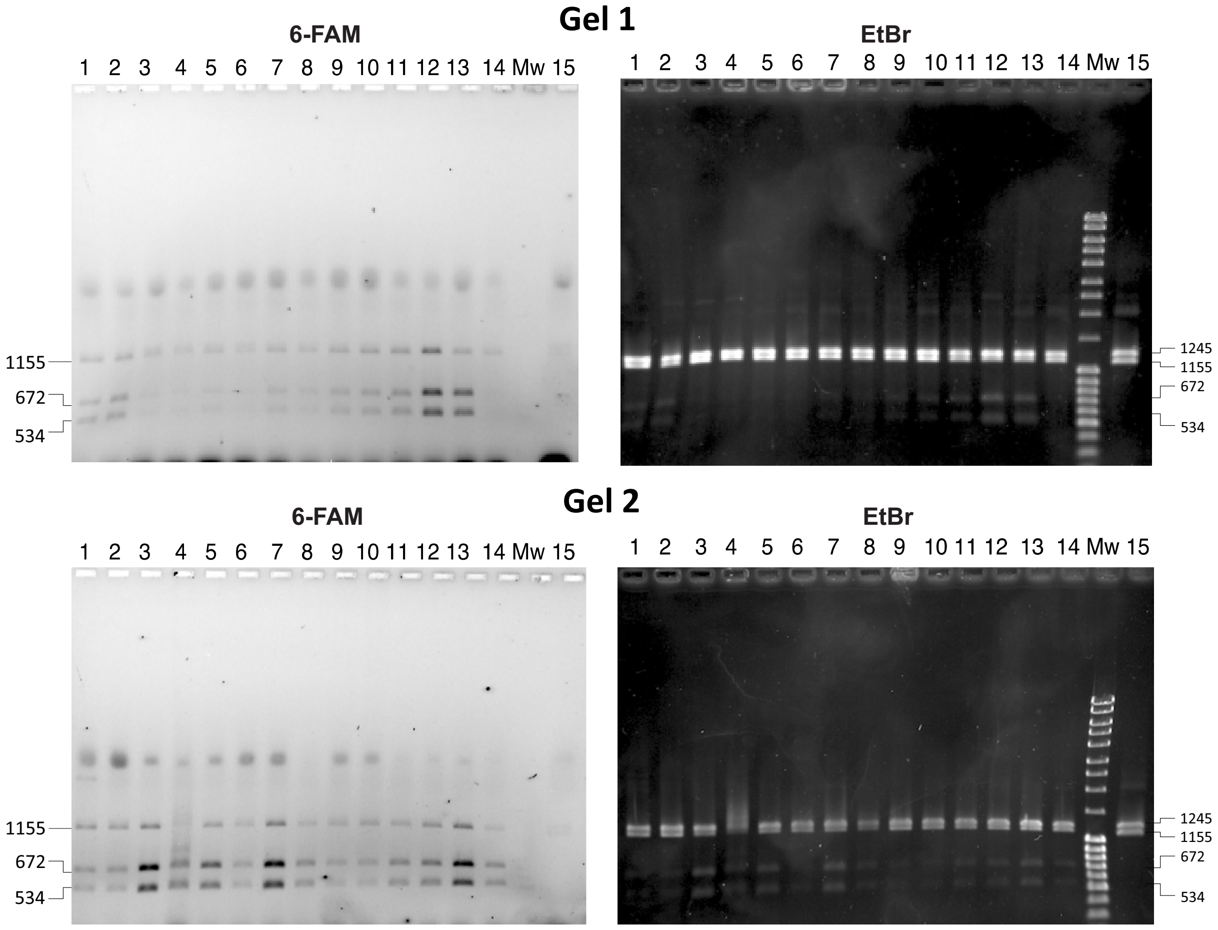

Gel 1

	Construct	'leader' / TSD segment
1	-16->+14	GAGCTCACTTCAAATCCGGGCGGCCCCACTACGAGGAG
2	-15->+14	TCCCAGAGCTCAAATCCGGGCGGCCCCACTACGAGGAG
3	-14->+14	CCAGAGCTCGCAAATCCGGGCGGCCCCACTACGAGGAG
4	-13->+14	TCCAGAGCTCGAAATCCGGGCGGCCCCACTACGAGGAG
5	$C_{-14}G$	TCCCAGAGCTGAAATCCGGGCGGCCCCACTACGAGGAG
6	$A_{-13}C$	TCCCAGAGCTCCAATCCGGGCGGCCCCACTACGAGGAG
7	$A_{-12}C$	TCCCAGAGCTCACATCCGGGCGGCCCCACTACGAGGAG
8	$A_{-11}C$	TCCCAGAGCTCAACTCCGGGCGGCCCCACTACGAGGAG
9	$T_{-10}A$	TCCCAGAGCTCAAAACCGGGCGGCCCCACTACGAGGAG
10	$C_{-9}G$	TCCCAGAGCTCAAATGCGGGCGGCCCCACTACGAGGAG
11	$C_{-8}A$	TCCCAGAGCTCAAATCAGGGCGGCCCCACTACGAGGAG
12	-24->+6	GACTCCGGTTCAAATCCGGGCGGCCCCACTGGTACCTG
13	-24->+5	GACTCCGGTTCAAATCCGGGCGGCCCCACGGTACCTGG
14	-24->+3	GACTCCGGTTCAAATCCGGGCGGCCCCGGTACCTGGAG
15	Δ-target	TGTCCAGAGCTCAATCCCCATGCTGGAGCACAAGACTG

Gel 2

	Construct	'leader' / TSD segment
1	-15->+14	TCCCAGAGCTCAAATCCGGGCGGCCCCACTACGAGGAG
2	$G_{-7}T$	TCCCAGAGCTCAAATCCTGGCGGCCCCACTACGAGGAG
3	$G_{-6}T$	GACTCCGGTTCAAATCCGTGCGGCCCCACGGTACCTGG
4	$G_{-5}T$	GACTCCGGTTCAAATCCGGTCGGCCCCACGGTACCTGG
5	$C_{-4}A$	GACTCCGGTTCAAATCCGGGAGGCCCCACGGTACCTGG
6	$G_{-3}T$	GACTCCGGTTCAAATCCGGGCTGCCCCACGGTACCTGG
7	$G_{-2}C$	GACTCCGGTTCAAATCCGGGCGCCCCCACGGTACCTGG
8	$C_{-1}G$	GACTCCGGTTCAAATCCGGGCGGGCCCACGGTACCTGG
9	$C_{+1}G$	GACTCCGGTTCAAATCCGGGCGGCGCCACGGTACCTGG
10	$C_{+2}G$	GACTCCGGTTCAAATCCGGGCGGCCGCACGGTACCTGG
11	$C_{+3}G$	GACTCCGGTTCAAATCCGGGCGGCCCGACGGTACCTGG
12	$A_{+4}T$	GACTCCGGTTCAAATCCGGGCGGCCCCTCGGTACCTGG
13	$C_{+5}A$	GACTCCGGTTCAAATCCGGGCGGCCCCAAGGTACCTGG
14	-24->+5	GACTCCGGTTCAAATCCGGGCGGCCCCACGGTACCTGG
15	Δ-target	TGTCCAGAGCTCAATCCCCATGCTGGAGCACAAGACTG

Supplementary Figure 3: Mapping of the insertion site of the LE26 oligonucleotide in plasmids harbouring mutated or partially deleted versions of the original target site of the *A. boonei* casposon. Plasmids were incubated at 37 °C for 80 min in the presence of 200 nM 6-FAM-labelled LE26 duplex and 100 nM His-tagged casposase as described in Materials and Methods. Reaction products were then digested with ApaLI, precipitated and analyzed by agarose gel electrophoresis. Gels were scanned for 6-FAM fluorescence and stained further with ethidium bromide (EtBr). ApaLI cleaves the target plasmids into two fragments of 1245 and 1155 ± 5 bp depending on the constructs, the latter bearing the target site. Single integration at the target site results in 6-FAM labelling of the 1155 bp fragment; linearization with tandem integration of two LE26 duplexes at the target site generates two labelled fragments of 672 and 534 bp from the 1115 bp fragment. The Mw lane of the ethidium bromide panel shows molecular size standards (Thermo Fisher) with sizes of 10000, 8000, 6000, 5000, 4000, 3000, 2500, 2000, 1500, 1031, 900, 800, 700 600, 500, 400, 300, 200, 100, and 80 bp. The sequence of the targets tested is shown below. Nucleotides belonging to the 5' leader are in highlighted orange; nucleotides belonging to the original TSD in yellow.

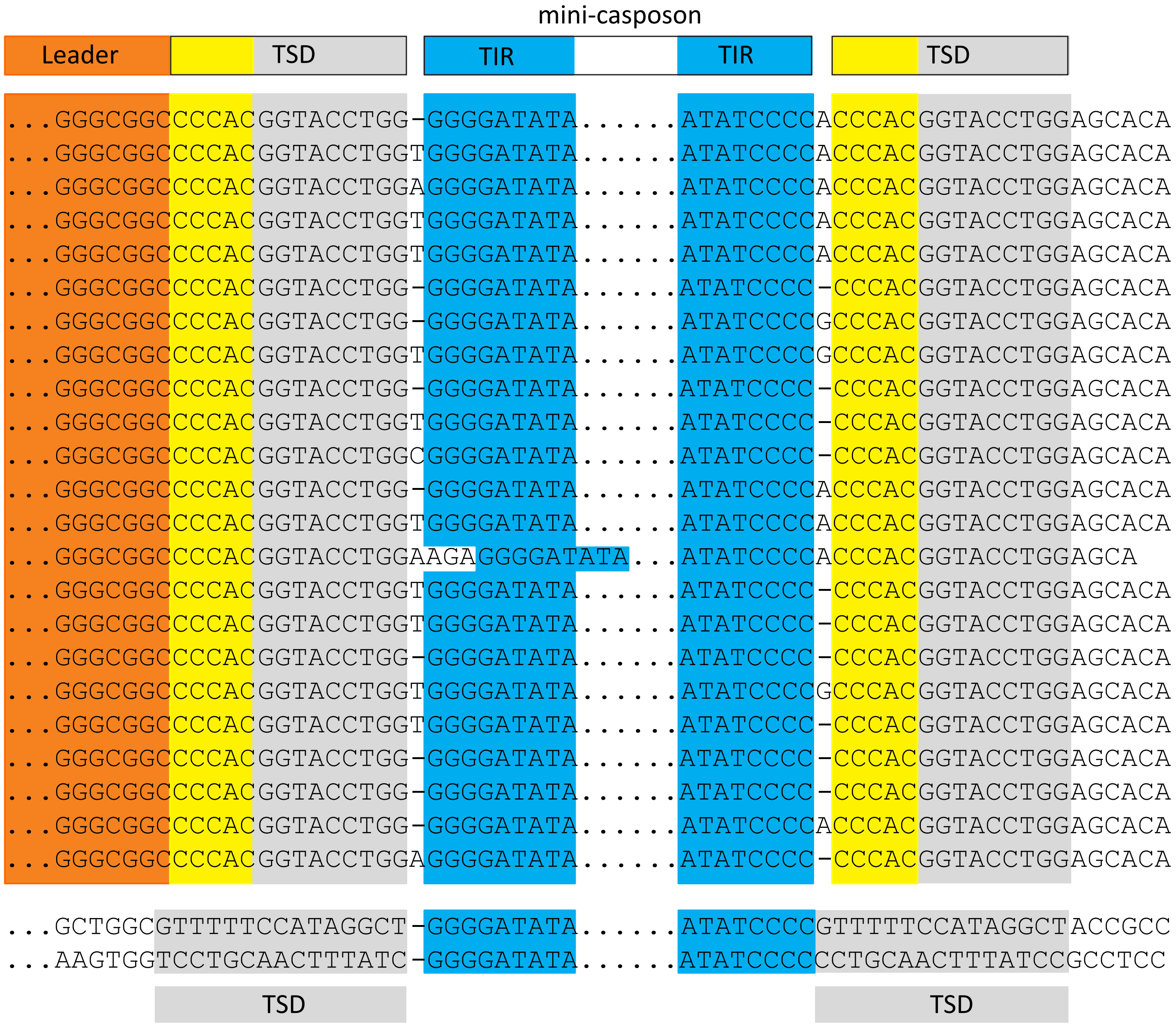

Supplementary Figure 4: Sequences bordering the left and right termini of the casposon encoding kanamycin resistance after insertion into the plasmid pMA-Targ 488-516, which carries nucleotides -24 to +5 of the original *A. boonei* target site. Nucleotides belonging to the leader are highlighted in orange; those belonging to the original TSD in yellow; those contributed by the vector to the TSD in grey; those belonging to the left and right TIR of the casposon in blue. Some clones contained an additional nucleotide between the TIR and the vector: this is most likely due to the terminal transferase activity of the Taq DNA polymerase, which was used to amplify the casposon substrate.
